# Diagnostic Accuracy of Magnifying Endoscopy with Narrow Band Imaging and Its Diagnostic Value for Invasion Depth Staging in Esophageal Squamous Cell Carcinoma: A Systematic Review and Meta-Analysis

**DOI:** 10.1155/2018/8591387

**Published:** 2018-05-20

**Authors:** Tingting Yu, Jin Geng, Wei Song, Zhonghua Jiang

**Affiliations:** ^1^Department of Gastroenterology Center, The No. 1 People's Hospital of Yancheng, Yancheng 224000, China; ^2^Department of Cardiology, Huai'an First People's Hospital, Huai'an 223001, China; ^3^Department of Gastroenterology Center, Huai'an First People's Hospital, Nanjing Medical University, Huai'an 223001, China

## Abstract

**Background and Goals:**

This study aimed to investigate the diagnostic accuracy of magnifying endoscopy with narrow band imaging (ME-NBI) and determine its value for invasion depth staging in esophageal squamous cell carcinoma.

**Methods:**

We searched the PubMed, Embase, and Cochrane Library databases and found relevant studies published up to December 2016. Quality Assessment of Diagnostic Accuracy Studies 2 was used to evaluate the quality of the studies. We calculated sensitivity, specificity, and positive and negative likelihood values from forest plots and determined summary receiver operating characteristic (sROC) curves for ME-NBI diagnostic accuracy analysis.

**Results:**

Ten studies met our criteria and were selected for this meta-analysis. A total of 1,033 patients underwent ME-NBI, and 207 of these patients received a diagnosis of staging mucosal or submucosal invasion. The pooled sensitivity, specificity, and positive and negative likelihood values of ME-NBI for the diagnostic rate were 0.90 (95% CI, 0.71–0.97), 0.90 (95% CI, 0.80–0.95), 6.74 (95% CI, 3.52–712.89), and 0.20 (95% CI, 0.10–0.42), respectively. The area under the curve (AUC) was 0.95 for all studies.

**Conclusions:**

ME-NBI provides a high diagnostic rate in evaluating the esophagus to diagnose squamous cell carcinoma. In the differentiation for invasion depth staging, ME-NBI was demonstrated to be superior to white light endoscopy and had a similar diagnostic rate compared with HF-EUS. However, HF-EUS had high positive likelihood values for invasion depth staging, suggesting that HF-EUS is a reliable method for confirming invasion depth staging.

## 1. Introduction

Esophageal cancer is the sixth leading cause of cancer-related deaths worldwide, causing 3.2% of all deaths [1]. The histologic type of esophageal cancer primarily comprises adenocarcinomas and squamous cell carcinoma. Squamous cell carcinoma accounts for approximately 90% of esophageal cancers in developing countries and Asian countries [2]. Esophageal squamous cell carcinoma (ESCC) infiltration that is limited to the mucosal membrane or submucosa is defined as superficial esophageal cancer (SESCC). The tumor invasion depth and lymph node metastasis have a direct effect on the prognosis of ESCC [3]. The depth of infiltration of ESCC is closely related to lymph node metastasis. Whereas ESCC invading the mucosa (T1a) (SESCC) shows a rate of lymph node metastasis of 5% to 9%, when these tumors invade the submucosa (T1b), the probability of lymph node metastasis ranges from 19% to 44% [4–6]. Therefore, early detection of ESCC is necessary to achieve a better quality of life and a better prognosis. As SESCC have a low rate of lymph node metastasis, for invasion depth staging, carcinoma infiltration that is limited to the mucosal membrane or submucosa is defined as “without invasion” and when carcinoma invades the submucosa, it is defined as “with invasion”.

Endoscopic methods for the diagnosis of ESCC include white light imaging endoscopy (WLI), magnifying endoscopy with narrow band imaging (ME-NBI), chromoendoscopy, and confocal endoscopy. In the early stage of esophageal cancer, mucosal changes are subtle and are easily missed by white light endoscopy because most SESCCs appear flat and/or isochromatic [7]. Chromoendoscopy methods such as Lugol staining can detect SESCCs with high sensitivity but low specificity, and the adverse reactions include severe discomfort and allergic reactions [8]. Additionally, the use of confocal endoscopy is not universal. Non-ME is a routine method for diagnosing the depth of invasion, and the diagnosis is based on the protrusion, depression, thickness, and hardness of the esophageal wall, which is subjective and tends to be affected by variability among observers. However, ME can clearly observe the microvascular structure, which is related to the development of esophageal cancer.

Advances in technology have led to improvements in image quality, rendering it easier to identify subtle changes that might occur in early stages of the ESCC. ME-NBI (Olympus Medical System Corporation, Tokyo, Japan) reported that, in patients at high risk for developing ESCC, ME-NBI can improve the diagnosis of esophageal neoplasia [7, 9, 10]. ME-NBI is an optical technology that involves the use of a narrow band optical filter to improve endoscopic diagnosis. The design of this filter corresponds to the peak absorption spectrum of hemoglobin to enhance mucosal and submucosal microvascular visualization [11]. SESCC lesions contain irregular microvessels, and the irregularity refers to the following changes in morphology: dilation, tortuosity, change in caliber, and various shapes (VS) [12, 13]. For example, there might be flat red changes and irregularities on the mucous membrane, which could easily be overlooked with a white light endoscope. Therefore, ME-NBI helps to accurately diagnose and reduce false-positive results when diagnosing SESCC [12, 14]. The advantage of ME-NBI is practicality; it requires only pressing a button and does not cause chest pain, and there is no risk of allergic reaction or pulmonary aspiration of the dye. Disadvantages include high equipment costs, which decrease accessibility.

Previous studies have demonstrated that lymph node metastasis is associated with the infiltration depth of ESCC [6, 15–18]. Therefore, accurate early diagnosis and correct staging before treatment are quite important to avoid unnecessary invasiveness and ensure a good quality of life for patients with ESCC [19–21]. Noninvasive imaging modalities such as computed tomography (CT) and magnetic resonance imaging (MRI) cannot clearly differentiate the layers of the esophageal wall. The use of ME-NBI in diagnosing the invasion depth of ESCC requires image enhancement and amplification but might lead to a rapid and objective diagnosis. Although HF-EUS is the most popular modality, it has produced conflicting results [22, 23]. Therefore, there is no consensus on the best method to assess the depth of invasion in ESCC patients.

The goal of the current study was to evaluate the accuracy of ME-NBI in the diagnosis of ESCC and to investigate the diagnostic value of ME-NBI in staging the invasion depth of ESCC.

## 2. Methods

### 2.1. Search Strategy

We searched the PubMed, Embase, and Cochrane Library databases for relevant studies published from 1980 to December 2016. The following groups of search terms were used to systematically search the literature: (A) esophageal cancer, magnifying endoscopy, narrow band imaging, and ME-NBI; (B) magnifying endoscopy, narrow band imaging, ME-NBI, and superficial esophageal cancer; (C) esophageal cancer, magnifying endoscopy, narrow band imaging, ME-NBI, and T1; and (D) esophagus, cancer, magnifying endoscopy, narrow band imaging, ME-NBI, and invasion depth. The search strategy was A or B or C or D. Our search was limited to human subjects. Two reviewers (Song Wei and Geng Jin) independently screened the title and summary of each article based on predefined inclusion and exclusion criteria. All of the articles that were ultimately selected were retrieved and reviewed by the same two reviewers (Song Wei and Geng Jin). The present study was conducted according to the MOOSE (Meta-analysis of Observational Studies in Epidemiology) recommendations. The protocol for our meta-analysis was registered in PROSPERO (International Prospective Register of Systematic Reviews, number 42017054707).

### 2.2. Inclusion Criteria

The study population comprised patients who underwent ME-NBI, some of whom had esophageal lesions that were suspected or confirmed to be ESCC based on endoscopic biopsy and examination such as ME-NBI. The intervention was an ME-NBI diagnosis for ESCC. Acceptable diagnostic methods included a histological evaluation and final pathological staging of specimens isolated during endoscopic submucosal resection (EMR), endoscopic submucosal dissection (ESD), or surgical resection. Acceptable studies were retrospective or prospective studies that included sufficient detailed results to allow for reconstruction of a diagnostic 2*∗*2 table (true positive, false positive, true negative, and false negative). In these studies, the diagnostic results of esophageal lesions examined by ME-NBI were compared with the results of the diagnostic methods described above.

### 2.3. Exclusion Criteria

Case reports and case series were excluded. Studies that did not provide sufficient data for reconstruction of a diagnostic 2*∗*2 table were deleted. We also excluded studies that were not available for magnifying endoscopy.

### 2.4. Data Abstraction

Two independent reviewers (Song Wei and Geng Jin) extracted the following data from the selected studies:

- Study characteristics: author (year), country, study design, patients, endoscopists, blinded pathologist, type of study
- Demographic characteristics: mean age, % male, population
- Interventions: equipment, lesions examined
- Outcomes: number of true-positive, true-negative, false-positive, and false-negative values.

#### 2.4.1. Outcomes for Analysis

The primary outcomes were the pooled sensitivities and specificities, positive and negative likelihood ratios, and diagnostic accuracy of ME-NBI for ESCC. Secondary outcomes included the pooled sensitivities and specificities, positive and negative likelihood ratios, diagnostic accuracy of ME-NBI, and other methods for staging the invasion depth of SESCC.

#### 2.4.2. Assessment of Study Quality

The quality of the reported studies is shown in Figure 2. The methodological quality of the studies was graded independently by two reviewers using the Quality Assessment of Diagnostic Accuracy Studies 2 (QUADAS2) tool [31]. Disagreement between the two extracting authors was resolved by consensus.

### 2.5. Statistical Analysis

Based on a comparison of an ME-NBI diagnosis with a final histopathological diagnosis by biopsy, EMR, ESD or surgical resection, we constructed 2*∗*2 statistical tables for each study in which 0 counts occurred in at least one cell of the study data, and a continuity correction of 0.5 was added to each value for that study. The true-positive, false-positive, true-negative, and false-negative values were then calculated. We also calculated the diagnostic rate of ME-NBI in invasion depth staging. Stata 12 statistical software was used to calculate the sensitivity, specificity, positive likelihood ratio (PLR), and negative likelihood ratio (NLR) for each study [32]. We used the bivariate binomial mixed model to pool the final sensitivity, specificity, PLR, NLR, and diagnostic odds ratio (DOR) [33]. A summary receiver operating characteristic curve (SROC) was constructed [34]. A SROC is similar to a standard ROC, the difference being that data from the SROC are derived from the sensitivity and specificity values in the individual studies. The area under the curve (AUC) of a SROC is a diagnostic indicator of performance. A test with an area under the curve (AUC) close to 1 is excellent, whereas a test with an AUC close to 0.5 is classified as poor [35]. *X*^2^ statistics, Cochran's *Q* test, and the *I*^2^ measure of inconsistency were used to evaluate heterogeneity [36–38]. Funnel plots were constructed to evaluate publication bias [39–41]. *P* < 0.05 was considered significant for all statistical methods.

Several factors were considered a priori as possible sources of heterogeneity:

1. Imaging modality: due to the presence of different disease types, a subgroup analysis was planned to assess the differences among disease types.
2. Different imaging equipment was GIF-Q240Z or GIF-FQ260Z, etc..
3. Study population: studies were performed in different countries.
4. Experience of endoscopists: endoscopists with more experience in recognizing abnormalities may yield better outcomes than individuals with less experience.

For invasion depth staging, we also performed a three-subgroup analysis: (1) ME-NBI, (2) HF-EUS, and (3) WLI.

## 3. Results

### 3.1. Literature Search

**Figure 1:**
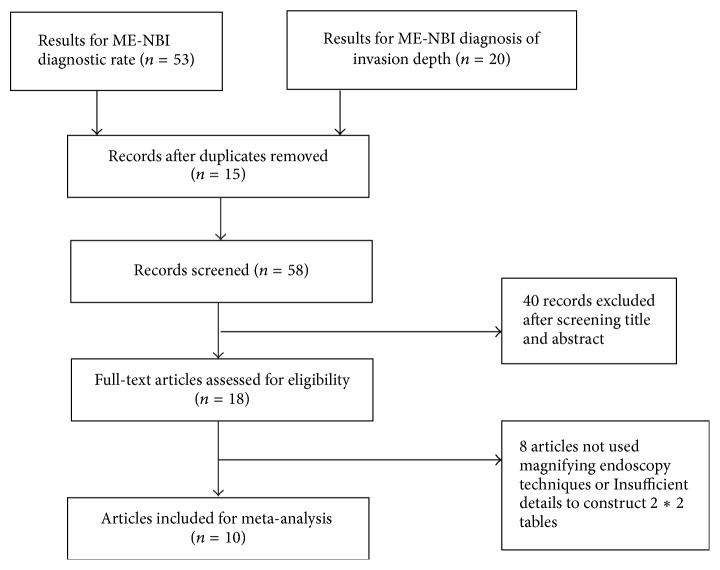
Flow diagram showing the study selection process.

**Figure 2:**
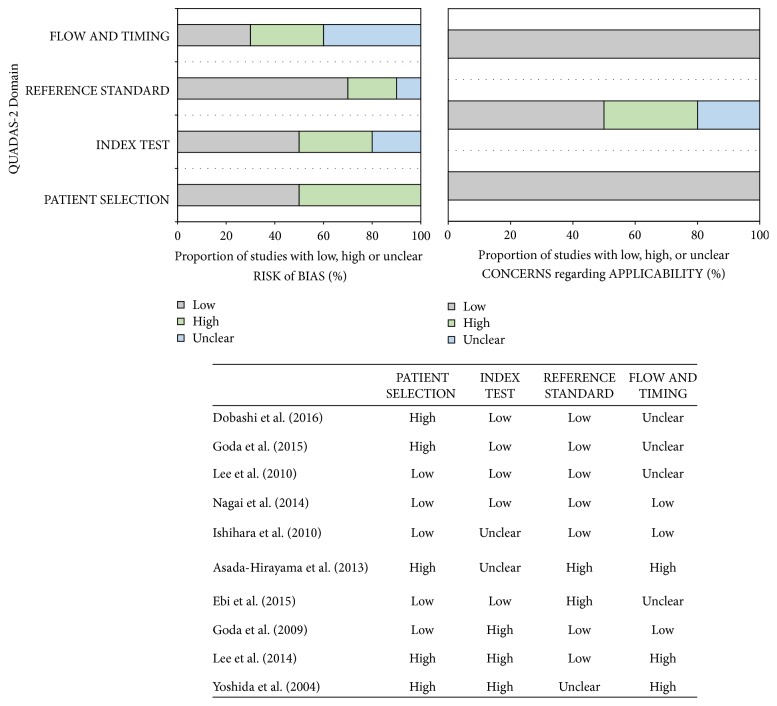
The Quality Assessment of Diagnostic Accuracy Studies 2 (QUADAS2) tool for the quality assessment of the eight studies included in the meta-analysis.

Using the search strategy, 73 documents were preliminarily identified, and 15 duplicates among those documents were excluded. After screening the titles and abstracts, 40 records were excluded for a variety of reasons (e.g., they were case reports, reviews, commentaries, or an animal study), which left 18 articles for evaluation. After examining the full texts of the articles, eight articles were excluded for not using magnifying endoscopy techniques or for having insufficient details to construct 2*∗*2 tables. Finally, 10 studies were selected for this meta-analysis [10, 12, 14, 24–30]. The procedure for the study selection is presented in Figure 1. There were seven studies from Japan [12, 14, 24–28], one study from the United States and Japan [29], one study from China [10], and one study from Korea [30]. In total, 1,033 patients underwent ME-NBI, and 207 of these received a diagnosis in terms of staging mucosal or submucosal invasion using histopathology. The characteristics of the studies are presented in Table 1.

### 3.2. ME-NBI Method

Diagnostic ME-NBI was performed in studies with a magnifying endoscope (GIF-H260Z or GIF-Q240Z; Olympus) and a 19-in high-resolution liquid-crystal monitor (OEV191H; Olympus).

### 3.3. Meta-Analysis

#### 3.3.1. Diagnostic Accuracy

**Figure 3:**
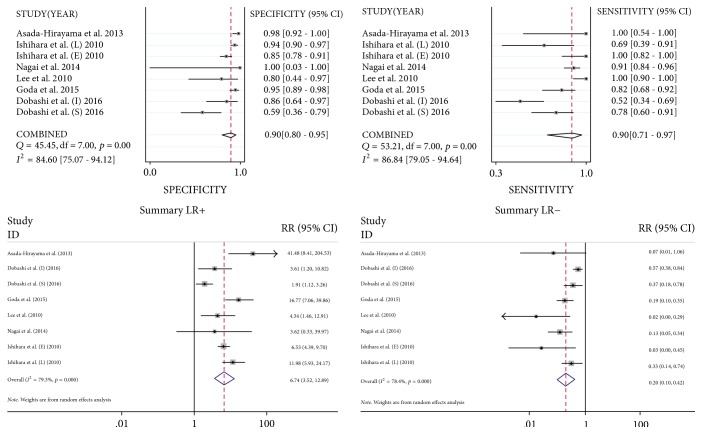
Forest plots of the sensitivity, specificity, PLR, and NLR of ME-NBI diagnostic accuracy for ESCC.

The Spearman correlation coefficient of ME-NBI for the diagnostic rate was 0.095 (*P* = 0.823), which indicated the absence of a diagnostic threshold effect.  Figure 3 demonstrates the sensitivity, specificity, PLR, and NLR of ME-NBI for the diagnostic rate. The pooled sensitivity and specificity of ME-NBI for the diagnostic rate were 0.90 (95% CI, 0.71–0.97) and 0.90 (95% CI, 0.80–0.95), respectively. The PLR and NLR were 6.74 (95% CI, 3.52–712.89) and 0.20 (95% CI, 0.10–0.42), respectively.

For invasion depth staging, four studies [12, 24, 27, 28] including 207 patients were analyzed for the diagnosis of ESCC. The Spearman correlation coefficient was −0.383 (*P* = 0.308), which indicated the absence of a diagnostic threshold effect. ME-NBI showed a sensitivity of 0.83 (95% CI, 0.69–0.92), a specificity of 0.85 (95% CI, 0.69–0.94), a PLR of 5.42 (95% CI, 1.96–14.97), and an NLR of 0.23 (95% CI, 0.12–0.47).

**Figure 4:**
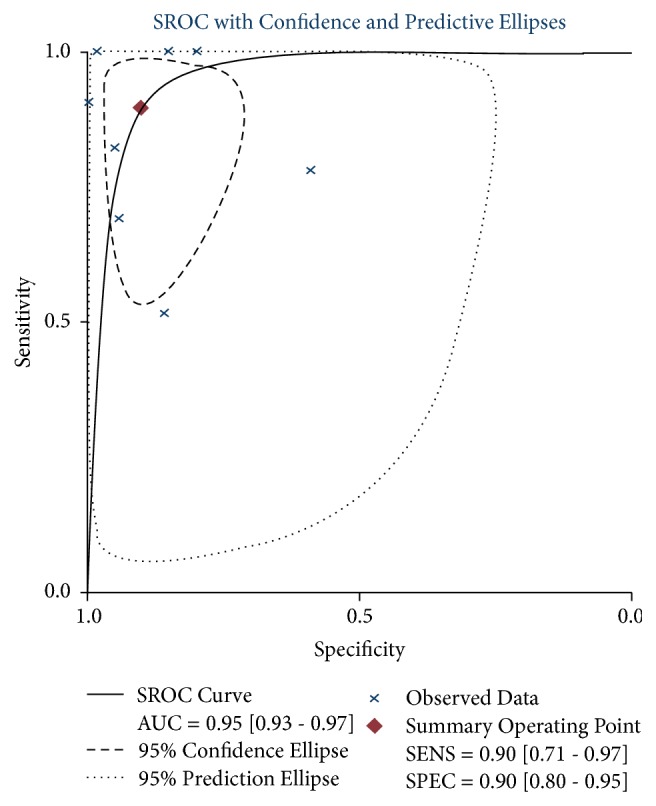
Summary receiver operating characteristic (SROC) curve of the ME-NBI diagnostic accuracy for ESCC. AUC, area under the curve.

SROC curves and the AUC demonstrated the accuracy of ME-NBI (Figure 4). For the diagnostic rate, ME-NBI showed an AUC of 0.95 for all studies.

#### 3.3.2. Metaregression

We conducted a series of univariate metaregressions to examine the relation between diagnostic yield and the following variables: disease type, country, endoscopists, method, and equipment. The outcomes of the regression analysis are presented in Tables 2 and 3. The disease type was statistically significant in the regression model for the diagnostic rate. In contrast, the equipment was statistically significant in the regression model for the staging of invasion depth. The results for the subgroup analysis for (1) ME-NBI, (2) HF-EUS, and (3) WLI are presented in detail in Figure 7.

#### 3.3.3. Small Study or Publication Bias Assessment

**Figure 5:**
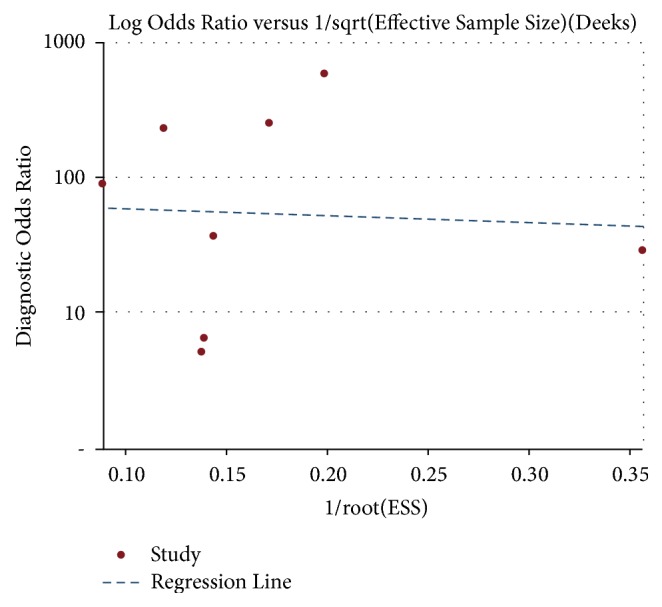
Funnel plots for the bias assessment of the ME-NBI diagnostic accuracy for ESCC.

**Figure 6:**
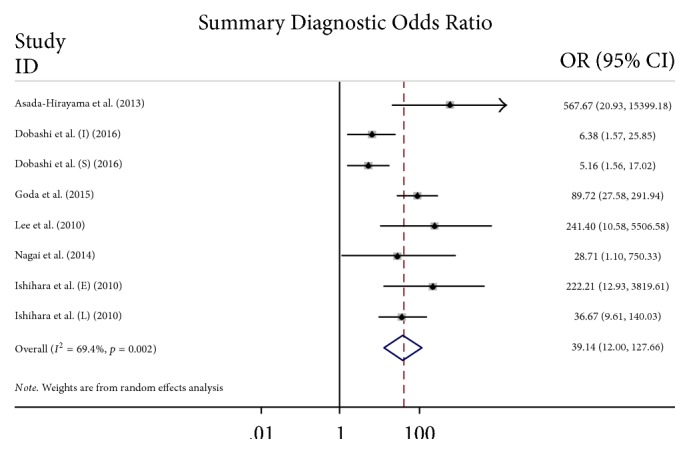
Forest plots of the DORs and 95% CIs of the ME-NBI diagnostic accuracy for combined studies reporting the ESCC diagnostic rate.

**Figure 7:**
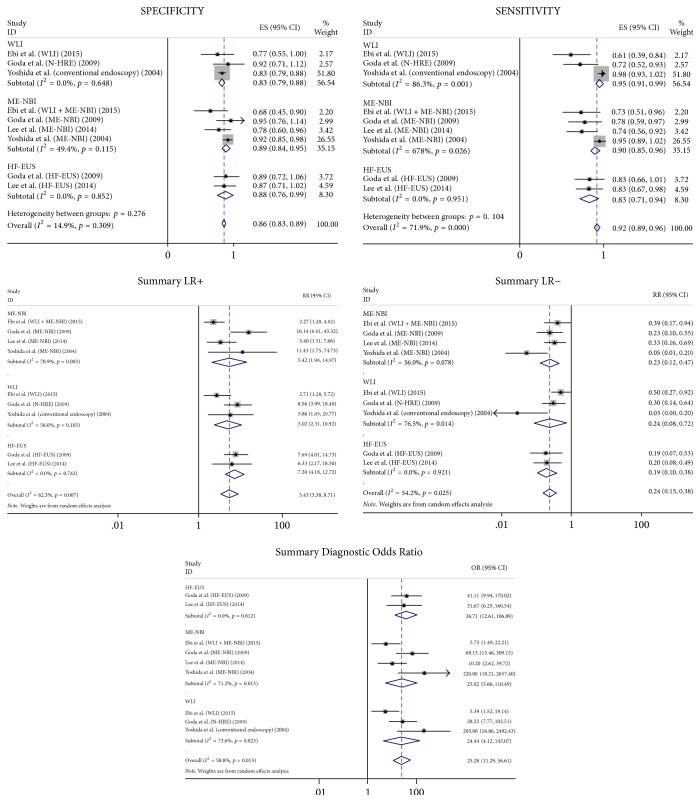
Subgroup analysis: forest plots of the sensitivity, specificity, PLR, NLR, and DORs of the ME-NBI, HF-EUS, and WLI for the diagnosis of invasion depth staging in ESCC.

The funnel plots for publication bias are presented in Figure 5, and *P* > 0.05 indicated no publication bias. Using the bivariate binomial mixed model, the DOR (Figure 6) for the combined studies for diagnostic rate was 39.14 (95% CI, 12-127.66).

## 4. Discussion

In ESCC, survival depends largely on the diagnostic stage. The five-year survival rate of patients in the first stage of disease is greater than 90%, whereas, in the third stage, this rate is approximately 10% [42]. In recent years, endoscopic minimally invasive treatments such as photodynamic therapy, argon plasma coagulation (APC), EMR, and ESD have provided options for the treatment of ESCC that is restricted to the mucosa. Compared with APC and photodynamic therapy, EMR and ESD surgical resection specimens can be evaluated histologically to assess the tumor infiltration depth, tumor-free margins, lymph nodes, and venous invasion as well as the degree of differentiation [6, 7]. Thus, early diagnosis and differentiation of invasion before surgery are important for determining the optimal treatment plan for patients with ESCC.

The current meta-analysis analyzed the accuracy of ME-NBI in the diagnosis and staging of ESCC and identified factors that might lead to heterogeneity across studies.

Clinical heterogeneity refers to studies with different standards (groups, study size, different research sites, etc.) and different gold standards for the diagnosis of disease. Methodological heterogeneity is caused by differences in the experimental design and study quality. Statistical heterogeneity is a combination of clinical and methodological heterogeneity among studies. We used histological findings as a standard. Therefore, the bias of the reference standard is low. However, there was high heterogeneity in our analysis, which might be due to variations in thresholds, countries, the doctors' experience, disease types, test methods, equipment, and the quality of the studies. Heterogeneity might primarily arise from study quality, although more high-quality data are required to explore this possibility. We identified two studies [14, 28] with the same lead author. However, one study was on the topic of diagnostic accuracy, and the other was on the topic of invasion depth. These two studies were also conducted during different time periods with different coinvestigators. For both, the accuracy in the diagnosis and staging of ESCC as well as the Spearman correlation coefficients were calculated, and the results indicated a *P* value < 0.05, indicating no threshold effect.

Overall, heterogeneity was present. Therefore, we used a bivariate binomial mixed model. In this study, we observed that ME-NBI for the diagnosis of ESCC showed sensitivity and specificity values of 0.90 and 0.90, respectively. For invasion depth staging, ME-NBI showed sensitivity and specificity values of 0.83 and 0.85, respectively. SROCs showed a trade-off between sensitivity and specificity. SROC curves were drawn to determine whether there was any heterogeneity among studies and to calculate the AUC. The results indicated that the diagnosis of ESCC by ME-NBI had high diagnostic performance with an AUC near 0.90. With PLR and NLR values of 6.74 and 0.20, respectively, ME-NBI showed high sensitivities and very low NLRs for the diagnosis of ESCC. The NLR assesses the ability of the test to exclude the disease in question. Thus, NLR < 0.1 provides strong evidence to rule out the disease [43], indicating that ME-NBI is a reliable modality for confirming ESCC.

For invasion depth staging, ME-NBI showed PLR and NLR values of 5.42 and 0.23, respectively. We also conducted a subgroup analysis to further explore the heterogeneity. In the differentiation for invasion depth staging, the ME-NBI was demonstrated to be superior to white light endoscopy and had a similar diagnostic rate compared with HF-EUS. However, HF-EUS had high PLRs (7.30) for the diagnosis of invasion depth staging. The PLR is a measure of how well the test identified the disease. Therefore, PLR > 0 provides strong evidence for a positive diagnosis [43] and suggests that HF-EUS is a reliable modality for confirming invasion depth staging.

Based on our results, we concluded that ME-NBI is associated with an increased rate of detection of ESCC. Therefore, a consensus regarding an education strategy for gastroenterologists and trainees is necessary for this technique to become more widespread.

### 4.1. Strengths and Limitations

This meta-analysis aimed to consider the increased rate in the diagnosis of ESCC using ME-NBI. We assumed an accurate pathological diagnosis in our results and discussion. Although the selected tests were considered to be statistically homogeneous, many different factors with the potential to introduce bias remained, such as study design, participant selection, different lesion types, and the experience of endoscopists. In particular, the accuracy of ME-NBI has been directly related to the experience of the endoscopists. Ishihara et al. [26] reported that their sensitivity was only 69% with less experienced endoscopists; this rate improved to 100% with experienced endoscopists.

Additionally, further high-quality studies are urgently required because the number of studies included in our meta-analysis was small. The statistical tests for small study effects/publication bias with a *P* value > 0.05 indicated no publication bias in our analysis. However, some studies with poor diagnostic performance or few patients might not have been published, and our search strategy only included published studies. Thus, well-designed prospective studies are required to provide stronger evidence.

## 5. Conclusions

ME-NBI provides a high diagnostic rate for the identification of ESCC patients. Another advantage of ME-NBI is that it does not require the application of chromoendoscopy, which can be difficult and might render the procedure more expensive. This meta-analysis analyzed the diagnostic abilities of the individual modalities. In fact, in clinical practice, the modalities are often used in combination. Thus, further studies are required to elucidate the benefits of binding patterns.

## Figures and Tables

**Table 1:** Characteristics of the included studies.

Author (Year)	Country	Treatment and comparison	Equipment	Patients	Lesions examined	Mean age	% Male	Population	Endoscopists	Blinded pathologist	Study design
Dobashi et al. (2016) [24]	Japan	ME-NBI (Simplified criteria)	GIF-H260Z; Olympus, 19-in high- resolution liquid-crystal monitor (OEV191H; Olympus)	147	54	67	88%	ESCC	2	Yes	Post hoc analysis
Goda. et al. (2015) [14]	Japan	LCE-PS (Lugol chromoendoscopy with pink-color sign) VS ME-NBI	GIF-H260Z; Olympus, 19-in high- resolution liquid-crystal (OEV191H; Olympus)	147	305	67	88%	SESCC	2	Yes	RCT (cross-sectional)
Lee et al. (2010) [10]	China	ME-NBI vs. conventional endoscopy	GIF-Q240Z or GIF-FQ260Z; Olympus	69	45	54	98.60%	HNC	4	Yes	RCT (crossover)
Nagai et al. (2014) [25]	Japan	ME-NBI vs. histologic diagnosis	GIF-H260Z; Olympus, 19-inch high-resolution liquid-crystal monitor (OEV19H, Olympus)	85	111	/	/	ESCC	/	Yes	RCT (crossover)
Ishihara et al. (2010) [26]	Japan	Experienced endoscopists vs. less experienced endoscopists	GIF-H260Z, Olympus	350	162 VS 186	66	82%	History of ESCC or/and HNC	5	Yes	RCT (cross-sectional)
Asada-Hirayama et al. (2013) [27]	Japan	ME-NBI vs. lugol chromoendoscopy	GIF-Q240Z or GIF-FQ260Z; Olympus	28	72	69	89%	ESCC or HGIN	/	Yes	Retrospective study

Invasion depth staging studies											
Goda. et al. (2009) [28]	Japan	N-HRE vs. ME-NBI vs. HF-EUS	GIF-2T240; Olympus; UM-3R; Olympus	72	101	65	86%	SESCC	3	Yes	RCT (cross-sectional)
Ebi et al. (2015) [29]	USA Japan	WLI vs. WLI + ME-NBI	GIF-Q260Z; Olympus	49	55	68	83.70%	SESCC	11	Yes	RCT (crossover) (multicenter, prospective)
Lee et al. (2014) [30]	Korea	ME-NBI vs. HF-EUS	GIF-H260Z; Olympus; GIF-2T240, Olympus; UM3D-DP20-25R, Olympus	45	46	66	93.30%	ESCC	1	Yes	Retrospective study

ESCC: esophageal squamous cell carcinoma, SESCC: superficial esophageal squamous cell carcinoma, HGIN: high-grade intraepithelial neoplasia, HNC: Head and neck cancer.

**Table 2:** Metaregression analysis of ME-NBI diagnostic accuracy.

Parameter	Estimate (95% CI)	Coef	*Z*	*P* > |*z*|
Sensitivity				
Disease type	0.81 [0.67–0.90]	1.43	−0.01	0.99
Country	0.84 [0.69–0.93]	1.69	−0.01	0.99
Doctor	0.91 [0.74–0.97]	2.33	0.90	0.37

Specificity				
Disease type	0.93 [0.82–0.97]	2.51	0.92	0.36
Country	0.91 [0.81–0.96]	2.31	0.61	0.54
Doctor	0.89 [0.78–0.95]	2.09	−0.71	0.48

Parameter	*I*-squared (95% CI)	LRTChi		*P* value

Joint Model				
Disease type	82.58 [63.08–100.00]	11.48		0.00
Country	63.02 [16.71–100.00]	5.41		0.07
Doctor	0.00 [0.00–100.00]	1.47		0.48

**Table 3:** Metaregression analysis of ME-NBI, HF-EUS, and WLI for the diagnostic accuracy of invasion depth staging in ESCC.

Parameter	Estimate (95% CI)	Coef	*Z*	*P* > |*z*|
Sensitivity				
Country	0.84 [0.73−0.91]	1.67	0.48	0.63
Method	0.83 [0.73−0.90]	1.60	0.09	0.93
Equipment	0.74 [0.59−0.85]	1.04	−2.06	0.04

Specificity				
Country	0.88 [0.80−0.93]	2.01	0.62	0.54
Method	0.87 [0.80−0.92]	1.93	0.10	0.92
Equipment	0.77 [0.68−0.84]	1.18	−3.49	0.00

Parameter	*I*-squared (95% CI)	LRTChi		*P* value

Joint Model				
Country	0.00 [0.00−100.00]	0.47		0.79
Method	0.00 [0.00−100.00]	0.03		0.99
Equipment	83.12 [64.35−100.00]	11.85		0.00
